# New Insights into the Treatment of Glomerular Diseases: When Mechanisms Become Vivid

**DOI:** 10.3390/ijms23073525

**Published:** 2022-03-24

**Authors:** Da-Wei Lin, Cheng-Chih Chang, Yung-Chien Hsu, Chun-Liang Lin

**Affiliations:** 1 Department of Internal Medicine, St. Martin De Porres Hospital, Chiayi 60069, Taiwan; orcaking88@gmail.com; 2 Department of Surgery, Chang Gung Memorial Hospital, Chiayi 613016, Taiwan; m7021@cgmh.org.tw; 3 Department of Nephrology, Chang Gung Memorial Hospital, Chiayi 613016, Taiwan; 4 Kidney and Diabetic Complications Research Team (KDCRT), Chang Gung Memorial Hospital, Chiayi 613016, Taiwan; 5 Division of Chinese Materia Medica Development, National Research Institute of Chinese Medicine, Taipei 613016, Taiwan; 6 Kidney Research Center, Chang Gung Memorial Hospital, Taipei 613016, Taiwan; 7 Center for Shockwave Medicine and Tissue Engineering, Chang Gung Memorial Hospital, Kaohsiung 833253, Taiwan

**Keywords:** minimal change disease, IgA nephropathy, membranous nephropathy, immunosuppressant, precision medicine

## Abstract

Treatment for glomerular diseases has been extrapolated from the experience of other autoimmune disorders while the underlying pathogenic mechanisms were still not well understood. As the classification of glomerular diseases was based on patterns of juries instead of mechanisms, treatments were typically the art of try and error. With the advancement of molecular biology, the role of the immune agent in glomerular diseases is becoming more evident. The four-hit theory based on the discovery of gd-IgA1 gives a more transparent outline of the pathogenesis of IgA nephropathy (IgAN), and dysregulation of Treg plays a crucial role in the pathogenesis of minimal change disease (MCD). An epoch-making breakthrough is the discovery of PLA2R antibodies in the primary membranous nephropathy (pMN). This is the first biomarker applied for precision medicine in kidney disease. Understanding the immune system’s role in glomerular diseases allows the use of various immunosuppressants or other novel treatments, such as complement inhibitors, to treat glomerular diseases more reasonable. In this era of advocating personalized medicine, it is inevitable to develop precision medicine with mechanism-based novel biomarkers and novel therapies in kidney disease.

## 1. Introduction

Glomerular disease is a disease of glomerular inflammation caused by immune-mediated damage to capillary endothelium, mesangium, or basement membrane. The clinical presentations of glomerular disease are diverse. Patients may have asymptomatic microscopic or gross hematuria and often develop proteinuria and even the severe as nephrotic syndrome. Glomerular diseases contributes to the prevalence of chronic kidney disease. It is the third leading cause of end-stage renal disease, resulting in dialysis and kidney transplantation and tremendous financial and medical burden [1].

Glomerular disease is classified by etiology or histopathology. Over the years, more detailed histopathological classification of individual glomerular disease has developed helpfully for diagnosis and prognosis. For example, the Oxford classification of IgA nephropathy does show prognostic value clinically, but it is still a descriptive categorization by the pattern of injury [2,3]. Nonetheless, selecting specific therapy based on the classification is still less desirable. Additionally, glomerular diseases are driven by many pathogenetic mechanisms. Therefore, the current classification systems cannot precisely determine which immunosuppressant or treatment can be best used against the disease. For decades, the main treatments for glomerulonephritis were extrapolated from other fields of medicine and became therapies of one-size-fits-all, with unpredictable responses and disease course. Thus, treatments for glomerular disease were once “art of trial and error”.

It is commonly known that the kidneys can filter about 150 L of blood in a single day. Therefore, kidneys are constantly exposed to blood-borne pathogens, toxic compounds, immunocomplex, and auto-antibodies. Innate immunity is essential for renal immune homeostasis, injury, inflammation, and repair as the first-line host defense. Furthermore, through the bridge of lymphocytes, the innate immunity can relay to the adaptive immune system. Consequently, with the booming trend in precision and personalized medicine, research in the immune pathogenesis of kidney diseases has sprung up in recent ten years. Here we first review the pathogenesis of three glomerular diseases, MCD, IgAN, and IMN, representing three significant types of glomerular diseases, namely, podocytopathy, immune complex associated glomerulonephritis, and kidney-focused autoimmune disease. In the last part, we explore the mechanisms of actions delivered by the current mainstay immunosuppressive drugs and the rationality for these medicine in treating glomerular diseases.

## 2. Minimal Change Disease and Primary Focal Segmental Glomerulosclerosis

Minimal change disease is the most common cause of the nephrotic syndrome. It frequently occurs in children older than 1 year old, accounting for 70–90% of the cases. This disease is often associated with dyslipidemia and hypercoagulability. After puberty, the incidence decreases significantly [4,5]. This disease is named by the absence of significant findings in glomeruli under the light microscope. Only foot process effacement by the electron microscope is noticed, without depositions of antibodies. Some cases show focal segmental glomerulosclerosis and are termed accordingly by that finding [6]. These pathological findings are denoted as podocytopathy, which includes diffuse mesangial sclerosis (DMS), minimal change disease (MCD), focal segmental glomerulosclerosis (FSGS), and collapsing glomerulopathy [7]. Minimal change disease and primary focal segmental glomerulosclerosis are thought of as a continuum of the same disease-podocytopathy. Evolution from minimal change disease to primary focal segmental glomerulosclerosis is possible over time [8,9,10,11,12]. DMS is rare, and collapsing glomerulopathy is regarded as a morphologic variant of FSGS [13]. 

**Figure 1:**
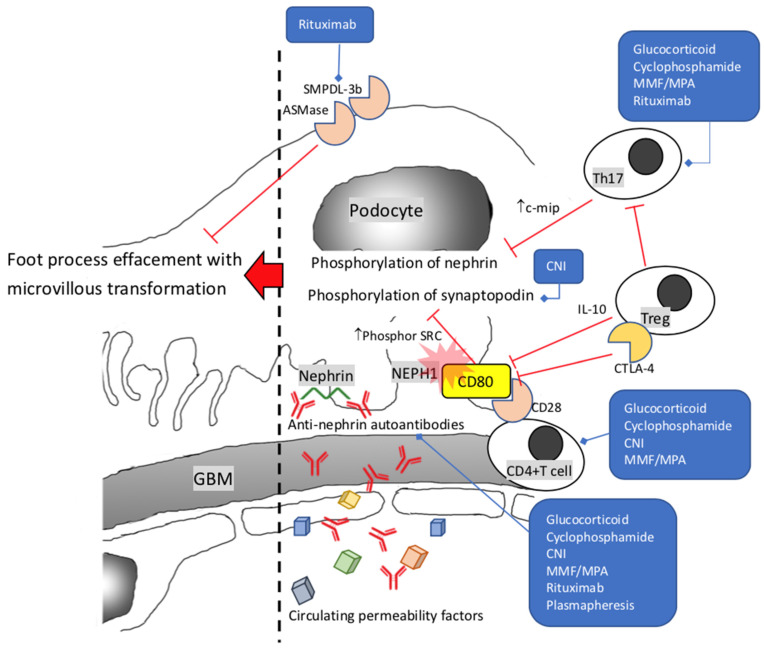
Pathogenesis of minimal change disease. Overexpressed CD80 and NEPH1 interact via extracellular domain. Interaction of CD80 and CD28 leads to increase expression of phosphor SRC, which causes dephosphorylation of synaptopodin. Increased expression of c-mip by Th17 results in dephosphorylation of nephrin. These activities and circulating permeability factors, including anti-nephrin autoantibodies, impair slit diaphragm integrity and destabilization of the actin-cytoskeleton. GBM: glomerular basement membrane; CNI: calcineurin inhibitor; MMF: mycophenolate mofetil; MPA: mycophenolic acid; SMPDL-3b: Sphingomyelin Phosphodiesterase Acid Like 3B; ASMase: Acid sphingomyelinase; c-mip: c-maf inducing protein; CTLA-4: cytotoxic T-lymphocyte-associated protein 4.

In 1974, Shalhoub [14] hypothesized that minimal change disease was caused by systemic dysregulation of T cells, which produced circulating factors to modify podocyte structures. Dysregulation of T cells and circulating factors lead to foot process effacement and proteinuria. The T-cell-associated hypothesis is based on observations from disease remission after measles infection, response to treatment with glucocorticoid and cyclophosphamide, and remission alone with the occurrence of Hodgkin’s disease and viral upper airway infection [15,16,17,18,19,20,21]. In 2011, Shimada [22] suggested that MCD was a ‘two hits’ disorder. First, the overexpression of CD80 on podocytes, possibly mediated with Toll-like receptor 3 (TLR3), is known to be induced by allergen, cytokine, or microbial products. Podocytes then behave like antigen-presenting cells [23,24,25,26,27,28]. These overexpressed CD80s first interact with NEPH1, disrupting slit diaphragms [25]. Second, T cell response is activated while CD80 binds to its receptor CD28 on T cells. This response can be terminated by CD80 binding to another receptor cytotoxic T-lymphocyte-associated protein-4 (CTLA-4), expressed by Treg (CD4+CD25+FoxP3+) [29,30]. Dysfunction of Treg to express CTLA-4 is the second hit [31]. Decreased expression of IL-10 by Treg, which inhibits the expression of CD80, is also noticed [32]. The binding of CD80 and CD28 in MCD increases the expression of phosphor SRC, which leads to dephosphorylation of synaptopodin and associated foot process injury [33]. Th17 is another subset of abnormally dysregulated T cells. In the animal models of adriamycin-induced nephrosis, Th17 is critically involved in the downregulation of phospho-nephrin and Bcl-2 by overexpressing c-maf inducing protein (c-mip). C-mip can inhibit Fyn-associated nephrin phosphorylation [34]. This inhibition leads to cytoskeleton disorganization with effacement of foot processes and apoptosis [35] (Figure 1). Besides this, protease-activated receptor-1 (PAR-1)-like mediator may exist in Th17 from MCD patients and induce a deleterious phenotypic change of podocyte via the JNK and p38 MAPK pathways [36]. A high Th17/Treg ratio is associated with increased proteinuria. Normalization in Th17/Treg ratio is seen if patients with MCD are sensitive to steroid treatment [37]. An earlier study proposed that the imbalance of Th1/Th2 cytokine activity is also related to the sensitivity to steroid treatment. Th2 activity dominance tends to be found in the steroid-sensitive primary nephrotic syndrome [38].

The implication of B cells in the pathogenesis of MCD is proposed by results of animal models and successful treatment by anti-CD20 monoclonal antibodies (i.e., Rituximab) [39,40]. While histopathological studies in renal biopsies show no deposit of antibody or immune complex, the role of B cells in MCD is much debated. Nonetheless, B cells can also act as antigen-presenting cells to T cells and secret cytokines and chemokines to support T cell activation [41,42,43,44,45,46]. An alternative explanation is that rituximab interacts with sphingomyelin phosphodiesterase acid-like 3b protein (SMPDL) on podocytes, stabilizes the actin skeleton, and prevents apoptosis [47,48,49]. SMPDL and acid sphingomyelinase (ASM) are also expressed by Th1 [50]. Referring to the effect of rituximab (RTX) on Th17 in treating rheumatoid arthritis to decline the Th17 and suppress IL-17 expression, RTX should also play a similar role in treating MCD [51,52]. 

Nonetheless, autoantibodies targeting nephrin in the slit-diaphragm junctional complex in animal models have been shown to cause podocyte injury and massive proteinuria [53,54,55]. In addition, anti-nephrin autoantibodies in both animal models and cultured podocytes result in the redistribution of nephrin away from the slit diaphragm along with the separation of intercellular junctions of adjacent podocytes [53,54,56]. This redistribution of the nephrin is similar to the findings in renal biopsies of patients with nephrotic syndrome [57]. Likewise, autoantibodies targeting nephrin are discovered in a subset of patients with noncongenital childhood and adult-onset MCD [58]. These autoantibodies are reduced or absent in the circulation during the treatment response and lead to massive proteinuria recurrence in allografts of renal transplantation. Successful treatment of this recurrence by plasmapheresis/rituximab is similar to the strategy for the rapid recurrence of primary FSGS in renal transplantation [59]. This discovery of anti-nephrin autoantibodies calls attention to the heterogenic pathogenesis of MCD and supports the hypothesis of the continuum from MCD to primary FSGS. 

Additionally, a few proposed circulating molecules are thought of as permeability factors, including cardiotrophin-like cytokine-1, soluble urokinase-type plasminogen activator receptor, and anti-CD40 antibody [60,61,62]. These factors lead to increase proteinuria and foot effacement. The exact roles of these circulating molecular need further investigation. 

After the initial immune-mediated insults, podocytes may undergo epithelial to mesenchymal transition (EMT). In a proinflammatory milieu, transforming growth factor (TGF)-β1 is upregulated. TGF-β1 suppressed the slit diaphragm-associated protein P-cadherin, zonula occludens-1, and nephrin and induced the expression of the intermediate filament protein desmin and interstitial matrix components fibronectin and collagen I. These events increase the permeability and proteinuria production by paracellular protein flux. TGF-β1 also promotes the expression of Snail, a key transcription factor in the initiation of EMT [63]. 

Nephrin and NEPH1 are key proteins of the constitutive component of the slit diaphragm and are critical for podocyte stability and integrity. The spatial arrangement of the extracellular domains of nephrin and NEPH1 provide integrity of slit-diaphragm while intracellular domains play a role in signaling network for modulation of actin-cytoskeleton changes in podocytes [64]. Phosphorylation of the nephrin tyrosine residues by Src family kinases, including Src, Fyn, Lyn, and Yes, plays a critical role in stabilizing the foot process and maintaining the slit-diaphragm structure [65,66,67]. This phosphorylation induces P85/PI3K binding and recruits Nck, leading to actin reorganization [68,69]. The reduced phosphorylation level of nephrin tyrosine residues is detected in MCD and MN, and phosphorylation stabilizes and restores podocyte foot process architecture [70,71,72]. On the contrary, phosphorylation of NEPH1 tyrosine residues is found in several podocyte injury models, and this can be assumed as a therapeutic target [73,74,75]. Another molecule, ephrin-B1, at the slit-diaphragm also involves the signaling network. Phosphorylation of ephrin-B1 leads to dissociation of nephrin and Par-6 from ephrin-B1 and promotes mobility of podocytes through activation of JNK [76]. Synaptopodin and α-actinin-4 (ACTN4) are other vital proteins that regulate the actin cytoskeleton in podocytes. Synaptopodin, associated with actin, can stabilize RhoA-mediated stress fiber formation in podocytes [77]. ACTN4 cross-links filamentous actin (F-actin) and supports the podocyte structure [78]. Phosphorylation by Src kinase makes dephosphorylation of synaptopodin serine/threonine residues leads to its degradation [79], while mutation of *ACTN4* is found in an autosomal dominant form of FSGS [78]. 

FSGS is the most common cause of end-stage renal disease among primary glomerular diseases in the united states [80]. Regarding genetic susceptibility, predisposing pathophysiological factors, and clinical courses, FSGS is categorized into five types. These include two common types (primary/idiopathic form and adaptive form) and three less common types (familial/genetic form, virus-associated form, and drug-induced form) [8]. While adaptive FSGS arises from overloaded processes involving increased single nephron GFR and intraglomerular hypertension, associated conditions include systemic hypertension, obesity, oligomeganephronia, very low birth weight, reflux nephropathy, unilateral renal agenesis, high protein diet, and any advanced renal disease with reduced functioning nephrons. Treatments for adaptive FSGS are aimed at the inhibition of the renin–angiotensin–aldosterone system to lower the glomerular filtration pressure. As human immunodeficiency virus (HIV) type 1, parvovirus B19, simian virus 40, cytomegalovirus, and Epstein–Barr virus are reported to induce virus-associated FSGS, predisposing drugs to drug-induced FSGS include heroin, interferons, lithium, pamidronate, sirolimus, calcineurin-inhibitor nephrotoxicity, and anabolic steroids. The main strategy is to stop or cure these exogenous insults for drug-induced FSGS and virus-associated FSGS. More than fifty genetic mutations expressed in podocytes or glomerular basement membranes are identified as the causes of genetic FSGS or steroid-resistant nephrotic syndrome [81,82]. These genetic/familial FSGS with single gene mutation usually express immunosuppressant resistance [83]. *APOL1* risk variant associated FSGS, which is found mainly in South African ancestry, shows many different characteristics than other genetic FSGS. Presented *APOL1* risk alleles confer susceptibility, but most subjects with two risk alleles may not develop kidney diseases. *APOL1* risk variant associated FSGS, once diagnosed, shows rapid progression to end-stage renal disease (ESRD) [84]. Not only FSGS, these *APOL1* risk variants much increase rates of hypertension-associated ESRD, HIV-associated nephropathy, end-stage of lupus nephritis, and other forms of non-diabetic kidney diseases. *APOL1* associated kidney diseases may be considered as an individual entity [85]. 

Pediatric nephrotic syndrome is known to respond well to steroid treatment. According to the KDIGO guideline for the glomerular disease of 2021, cyclophosphamide or oral levamisole is the first-line alternative therapy for steroid-sparing regimens. Other drugs, such as mycophenolate mofetil, a calcineurin inhibitor, or rituximab, can be used as second-line treatment. Calcineurin inhibitor as the initial second-line therapy for steroid resistance nephrotic syndrome is recommended. Genetic testing to exclude congenital/familial nephrotic syndrome or genetic disorder and renal biopsy for cases with steroid resistance, familial history of steroid-resistant nephrotic syndrome/FSGS, or syndromic features when classifications cannot be judged by clinical assessments [86]. The steroid is the first-line treatment for primary FSGS, and calcineurin inhibitor is the choice with steroid resistance. Calcineurin inhibitors can be replaced by mycophenolate combined with high-dose dexamethasone, rituximab, or ATCH if steroids are intolerant [87]. While comparing with minimal change disease, there is higher likelihood of steroid resistance and a higher rate of progression into end-stage renal disease in primary FSGS. The rate of glucocorticoid-induced remission is lower in primary FSGS. A portion of steroid-sensitive nephrotic syndrome associated with minimal change disease may become steroid resistant later as repeated renal biopsies often reveal a change of focal segmental sclerosis due to persistent and repeated podocyte injury with associated podocyte loss beyond the point of no return [12,88].

## 3. IgA Nephropathy

IgA nephropathy (IgAN) is the most common glomerulonephritis. The incidence of IgAN is high in Pacific-Asian regions. Familial clustering is noticed in an area where offspring often own a common ancestor [89]. Although IgAN occurs sporadically, about 5–8% of patients have relatives with biopsy-proven IgA or abnormality in urine. Patients with familial history of IgAN often have a worse prognosis [90]. However, the clinical presentations of IgA are inconsistent. Patients may be diagnosed simply due to asymptomatic hematuria or progressive renal function decline. Nephrotic range proteinuria is also commonly found. Although rapid progression to renal failure is rare, this disastrous phenotype still could happen with the crescent formation of more than 50% glomeruli [91]. 

**Figure 2:**
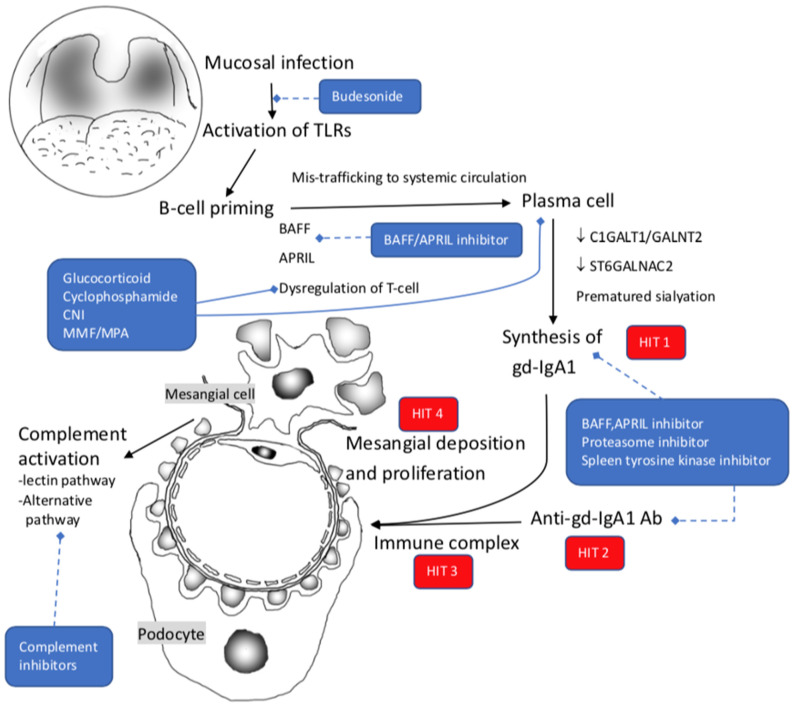
Pathogenesis of IgA nephropathy with 4 hits theory. Hit 1: synthesis of gd-IgA1; Hit 2: anti-gd-IgA1 antibody production; Hit 3: Circulating IgA immune complex formation; Hit 4: Circulating IgA immune complex deposition to the glomeruli. TLRs: Toll like receptors; BAFF: B-cell activating factor; APRIL: a proliferation-inducing ligand; gd-IgA1: galactose-deficient IgA1; C1GALT1: Core 1 Synthase, Glycoprotein-N-Acetylgalactosamine 3-β-Galactosyltransferase 1; GALNT2: Polypeptide N-Acetylgalactosaminyltransferase 2; ST6GALNAC2: ST6 N-Acetylgalactosaminide α-2,6-Sialyltransferase 2; anti-gd-IgA1 Ab: anti-gd-IgA1 antibody; CNI: calcineurin inhibitor; MMF: mycophenolate mofetil; MPA: mycophenolate acid. Blue dash lines denote undergoing novel treatment in IgA nephropathy.

The most acceptable pathogenic paradigm of IgAN is the multiple-hit pathogenesis model (Figure 2) [92]. First is a defect in the regulation of IgA1production and glycosylation. IgA1, a predominant subclass of IgA in serum, is characterized by the insertion of two octapeptide repeats in the hinge region between Cα1 and Cα2 domains. This hinge region is absent in IgA2 [93,94]. This hinge region is rich in Ser, Thr, and Pro residues which are potential sites for O-glycosylation with three to six in the amount [93,95,96]. These O-glycans all have an N-acetyl galactosamine core (GalNAc core) attaching to Ser/Thr residuals. These GalNAc cores can exist alone or extend with β 1–3 linked Gal to form disaccharides. These disaccharides can further be sialylated on GalNAc, Gal, or both sugars [97,98]. IgA with abnormal, defective galactosylation of O-glycans is found in patients with IgA nephropathy [99,100]. Studies from IgA-producing cells in peripheral blood suggest that premature sialyation may lead to this abnormal IgA glycosylation [101]. This galactose-deficiency IgA(gd-IgA) can circulate as a monomer or in self-aggregated form. The level of gd-IgA1 in circulation may be partially influenced by exogenous factors, such as bacteria-deprived proteases [102]. It is supposed that the environment, food antigens, or mucosal infections directly or indirectly through TLR signaling trigger the maturation of these B cells. For example, ligation of TLR-9 in B cells with bacterial DNA leads to polyclonal B cell activation, immunoglobulin production, and class switching [103,104]. These signaling increase expression of B cell activation factor (BAFF) and a proliferation-inducing ligand (APRIL) signaling, which co-stimulate B cells at lamina propria maturing into plasma cells [105,106,107,108,109]. These primed B cells, which secret mucosal IgA, are mis-home to circulation where gd-IgA1 immune complexes formed. With circulation, these mis-home B cells can migrate to bone marrow and kidneys and form a tertiary lymphoid organ with focal proliferation [110,111,112]. 

TLR7 was expressed in abundant these infiltrated CD19+ B cells and is closely related to renal function and histopathological findings. The MyD88 dependent signaling pathway promoted B cell expansion with immunoglobulin secretion and synthesis cytokines (IL-6, IL-12, and IL-1β). IL-6 leads to mesangial proliferation, apoptosis of podocytes, endothelial dysfunction, extracellular matrix production, and renal fibrosis, while IL-12 can recruit and accumulate lymphocytes and induce the secretion of IFN-γ [113]. 

Disorder of enzyme expression via TLR signaling pathways plays a crucial role in synthesizing gd-IgA1. Overexpression of TLR7 leads to more Polypeptide N-Acetylgalactosaminyltransferase 2(GALNT2) proteins [113]. GALNT2 is the critical determinant of the numbers and pattern of O-glycans to the hinge of IgA1. Glycoprotein-N-acetylgalactosamine 3-β-galactosyltransferase 1(C1GALT1) is responsible for adding galactose to GalNAc via its core 1 β 3-Gal-T-specific molecular chaperone (COSMC). Low C1GALT1/GALNT2 ratio in IgAN with overexpression of GALNT2 leads to higher gd-IgA production. Ligation of TLR-4 in B cells with bacterial PLS can also decrease the activity of C1GALT1 and associated defective galactosylation by methylation of the Cosmc gene [114]. Another similarly reduced expression is activated by TLR9, with a synergy of IL-6 and APRIL to affect the O-glycosyltransferase ST6GALNAC2 [115]. 

Once the gd-IgA is formed, the second hit is the formation of anti-glycan antibodies. The exposed GalNAc mimics bacterial or viral antigens and can be recognized by specific anti-glycan antibodies. These anti-glycan antibodies lead to the formation of the circulating immune complex [116,117]. Normal IgA1 has a short half-life of about five days and is rapidly catabolized by hepatocytes via sialoglycoprotein receptors (ASGP-R) [118]. However, if the sialic acid is linked to GalNAc or IgA is bound with antibodies, the clearance by hepatocytes may be hindered. Serum gd-IgA1 is bound with antibodies as an immune complex. That is why gd-IgA1 often remains in circulation for a prolonged period. From animal studies, circulating polymeric complex is known to induce cleavage of the extracellular domain of FcαR (CD89) and form an IgA1-CD89 complex (hit 3). It can further precipitate in mesangial deposition with high affinity [119]. 

While depositing in the mesangium, these immune complexes stimulate mesangial proliferation and production of IL-6 and TGF-β, which recruit leukocytes with inflammatory reactions and promote glomerular and interstitial fibrosis (hit 4) [120,121,122]. Additionally, activating the complement system involves glomerular inflammation. C3 glomerular staining can be found in over 90–95% of biopsies, while C1q is generally negative. Such dichotomy suggests activating the alternative pathway, likely the lectin pathway [123]. Positive C4 staining with the absence of C1q is found in 40% of biopsies, while mesangial deposition of mannose-binding lectin (MBL) is about 25%. Additionally, L-Ficolin and MBL-associated serine protease are found in positive MBL staining biopsies. These findings suggest the activation of the lectin pathway, and patients having this pattern suffered from more severe histologic damage and more proteinuria. Proteins, including factor B, factor H, and factor H related proteins (FHR), of the alternative pathway, were also found in some biopsies. Accumulating data implied that, contrary to factor H terminating activation, FHR-1 and FHR-5 amplify alternative pathway activation by competing with factor H. Elevation of FHR1 and FHR5 in circulation are correlated with the activity of IgAN, and high FHR5 level is associated with poor response to immunosuppressant [124,125]. 

Defective antigen handling by mononuclear cells in peripheral blood of patients with IgAN increases the expression of C-X3-C Motif Chemokine Receptor 1 (CX3CR1). Meanwhile, glomerular and urinary fractalkine, the ligand of CX3CR1, in patients with IgAN is also present in a high amount. Therefore, transmigration of these CX3CR1+lymphocytes into the tissue will damage the endothelial cell and role in vascular injury and hematuria [126]. 

Genome-wide association studies (GWAS) reveal, in contrast to the other two primary glomerular diseases, a highly complex polygenic architecture in IgA nephropathy with nearly 20 genome-wide significant loci of minor to moderate effects. Like other major immune-mediated glomerular diseases, the MHC locus on chromosome 6p21 strongly correlates with genetic susceptibility. HLA-DQA1*0101 and HLA-DQB1*0301 appear as risk alleles, while HLA-DQA1*0102 and HLA-DQB1*0201 reveal to be protective [127]. Additionally, the discovery of non-MHC loci reinforced the roles of innate and adaptive immunity in pathogenesis. The *HORMAD2-LIF-OSM* locus encodes two mucosal immunity and inflammation cytokines, and the IgAN risk allele in this locus has shown a concordant effect on the risk of tonsillitis and tonsillectomy [128,129]. The *TNFSF13* locus encodes APRIL involving the regulation of IgA production [130]. *DEF* locus encodes human antimicrobial peptides called α-defensins 1 and 3 (*DEFA1* and *DEFA3*), and a low copy number increases the risk for IgAN [131]. CARD9 is a proinflammatory molecule promoting the activation of the NF-κB pathway, and this risk variant provides the genetic evidence of the NF-κB pathway in the pathogenesis of IgAN [127]. The discovery of complement system-related loci includes a variant on chromosome 1q32 at the *CFH* locus and the *ITGAM-ITGAX* locus [127,128]. Deletion of *CHF1* and *CHF3*, which encode factor H-related peptides, show protection to enhance the effect of factor H in the alternative pathway. *ITGAM* and *ITGAX*, fixed in East-Asian populations, encode leukocyte-specific integrin αM and αX of complement receptor 3 and 4 involving the adhesion, migration of leukocytes, and phagocytosis of macrophages. GWAS also reveals quantitative endophenotypes in IgAN. Variants with lower expressions of *C1GALT1* encoding core 1 synthase, glycoprotein-N-acetylgalactosamine 3-beta-galactosyltransferase 1, and *C1GALT1C1* encoding COSMC lead to increased production of gd-IgA1 [132]. Genetic studies can provide a powerful tool to detect the earliest molecular participating factors of diseases and facilitate improved disease classification with complex traits based on molecular mechanisms. However, current GWAS results of IgAN are limited to East-Asian and European cohorts and can only explain 7% of the disease risks. More diverse populations and the discovery of more risk variants are expected.

Although the immune system prominently involves the pathogenesis, clinical results from the immunosuppressant treatment are not inspiring. Glucocorticoids in several small trials showed a reduction in proteinuria but no benefit in remission rate. In the Supportive Versus Immunosuppressive Therapy of Progressive IgA nephropathy (STOP) IgAN trial, the treated group received glucocorticoid as monotherapy while GFR ≥ 60 mL/min per 1.73 m^2^ or glucocorticoids plus cyclophosphamide for three months and azathioprine from month 4 to 36 if GFR = 30–59 mL/min per 1.73 m^2^. Although with a significant reduction in proteinuria production, there was no effect on the decline of renal functions [133]. A retrospective analysis in Europe reported glucocorticoid treatment significantly reduces proteinuria with better renal survival, even if eGFR ≤ 50 mL/min per 1.73 m^2^, and the benefit is positively correlated to the level of proteinuria. The prognostic predictivity of the MEST score was blunted after immunotherapy [134]. The Therapeutic Evaluation of Steroids in IgA Nephropathy Global (TESTING) Study assigned patients with proteinuria more than 1 g/day to symptomatic treatment or oral methylprednisolone. The benefit could be seen in the treated group with better primary endpoints (death, ESRD, or decline in renal function). Still, the study was interrupted early owing to excessive infectious events [135]. Small trials with Mycophenolate mofetil and hydroxychloroquine revealed mixed results in Asian groups [136,137]. A meta-analysis of RCTs for treatment with the mycophenolate mofetil in IgA showed no additional benefit or adverse events than other immunosuppressants [138]. Even rituximab, a monoclonal antibody against CD20+ B cells, does not improve proteinuria and renal function [139]. Some studies interceded with rituximab for invalid against plasma cells which produce the gd-IgA predominantly [140,141]. There are ongoing novel agent trials, including budesonide (non-absorbable steroid, targeting the intestinal mucosal immune system) [142], adrenocorticotropic hormone (synthetic ACTH) [143], Blisibimod (NCT02052219, withdrawn), and atacicept (NCT02808429, targeting BAFF and APRIL signaling pathways), bortezomib (Proteasome, targeting plasma cells) [144], and complement inhibitors (C5a receptor inhibitor Avacopan, NCT02384317; Eculizumab [145], a humanized monoclonal antibody that blocks C5 activation). Although a retrospective cohort analysis in Japan revealed benefits from tonsillectomy for IgAN, with improved renal survival rate, all need more RCTs for validating their therapeutic effects [146].

## 4. Membranous Nephropathy

Membranous nephropathy (MN) occurs at all ages but predominantly among males, with a mean age of diagnosis at the fifties and sixties. It is the first leading cause, other than diabetes, of nephrotic syndrome in Caucasian adults [147,148]. About four-fifth is idiopathic, and the remaining cases are secondary to medications or other diseases, such as systemic lupus erythematosus, viral hepatitis, or malignancies. About one-third of the patients with pMN undergo spontaneous remission. Nonetheless, it is still the second and third leading cause of end-stage renal disease amid primary glomerular diseases in America and Europe, respectively [149,150]. 

MN is an autoimmune disease, presenting with immune complex deposition between the podocyte and the glomerular basement membrane. With the damage of integrity of podocytes, a large amount of protein is lost in the urine. Heavy proteinuria brings about hypoalbuminemia, anasarca, and hypercoagulability. With persistent proteinuria, about 40–50% of patients develop renal failure within ten years. 

During the 1970s, MN was supposed to be an immune complex associated with nephropathy. Until 2002, the first antigen of MN in humans, neutral endopeptidase (NEP), was discovered from a case of congenital MN [151]. By mass spectrometric analysis of electrophoretic gel band from the serum of patients with MN, M-type phospholipase A2 receptor (PLA2R) was identified. Autoantibodies against PLA2R can be identified in 70–80% of patients with primary MN [152]. PLA2R is a transmembrane glycoprotein, present in large amounts on the apical surface of podocyte processes and may be shed into urine. Its specific function in podocytes is unclear. It is known that group IB secretory phospholipase A2(sPLA2 IB) via PLA2R is toxic and can induce human podocyte apoptosis [153]. Auto-antibodies, without epitope spreading, targeting the N-terminal cysteine-rich (CysR) domain of PLA2R only contribute better prognosis than those with epitope spreading to C-type lectin-like domains (CTLD) [154,155]. Thrombospondin type 1 domain-containing 7A(THSD7A) is another multidomain transmembrane glycoprotein identified by mass spectrometry. THSD7A is found in 2–3% of primary membranous nephropathy (pMN) [156]. By similar model and techniques, other candidates, including EXT1/EXT2 [157,158], NELL1 [159], SEMA3B [160], NCAM1 [161], PCDH7 [162], and HTRA1 [163] were found. Some of them are found concomitantly in the lupus MN or malignancy-associated MN simultaneously, and some remain lack the corresponding antibody. Their roles as real antigens or as biomarkers are still controversial. 

Earlier studies suggested that dysregulation in immune phenotype, characterized by a decreased Treg number and increased plasma cell/regulatory B cells, is associated with pMN [164]. Predisposing factors to autoantibody formation include genetic susceptibility (e.g., variant in *HLA-D*), or alternation in antigen expression by exogenous factors (e.g., air pollution, infections) [165,166,167,168]. The most extensive multi-ethnic genome-wide association study (GWAS) in MN found significant loci with genetic effects encoding two transcriptional master regulators of inflammation (i.e., *NFKB1* and *IRF4*). These findings underscore the effects of transcriptional regulation in an inflammatory response and the implication of the role of infection in disease induction [168]. In China, a study found that long-term exposure to air pollution increases the incidence of MN [169]. Like NEP as a trigger to induce allo-autoantibody, associated MN has been found in a case of Pompe disease who received enzyme replacement therapy and developed an alloimmune response to the recombinant human arylsulfatase B (rhASB) [170].

**Figure 3:**
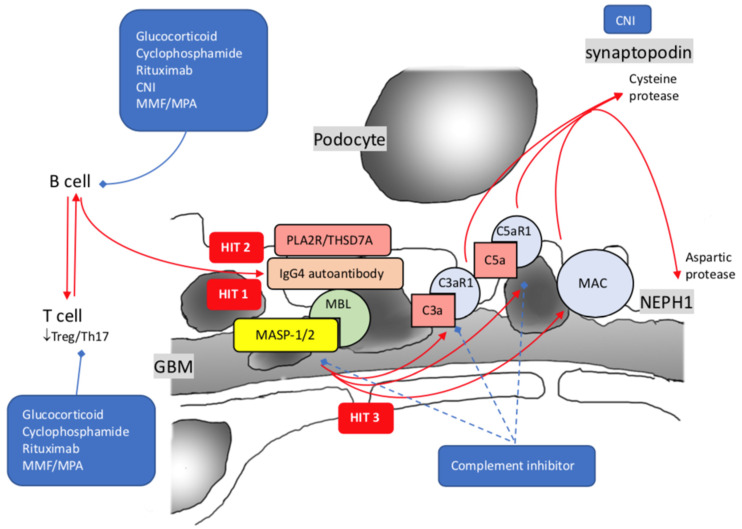
Pathogenesis of idiopathic membranous nephropathy with 3 hits theory. Hit 1: Synthesis of autoantibodies against PLA2R/THSD7A; Hit 2: IgG4 predominant autoimmunization against PLA2R/THSD7A by class shift; Hit 3: Increased expression of C3a1 and C5a1 alone with MAC. PLA2R: Phospholipase-A2-Receptor; THSD7A: Thrombospondin type I domain-containing 7A; MBL: mannose-binding lectin; MASP-1/2: mannose-associated serine protease 1 and mannose-associated serine protease 2; MAC: membrane attack complex; CNI: calcineurin inhibitor; MMF: mycophenolate mofetil; MPA: mycophenolate acid; blue dash lines imply possible novel treatments.

The framework of injury to podocytes with proteinuria production unveiled the prelude by precipitation of subepithelial immune deposits, followed by activation of the complement system and assembly of the terminal complement component C5b-9. IgG and C3 are present in disease-established cases, but C1q is usually weak or absent, as observed by immunofluorescence staining, implying the minor role of the classical complement pathway [171]. As the disease progress, the IgG subclass switch from IgG1 and IgG3 at the initial stage to enriched IgG4 at the last stage, activation of the complement system may also have a similar switch from the classical to the alternative or lectin pathway. Besides that the complement membrane attacks complex C5b-9, the upregulation of C3a and C5a receptors (C3aR1 and C5aR1) is shown on podocytes of pMN. There is a positive correlation between urinary C5a and the level of anti-PLA2 antibodies or proteinuria [172,173,174,175]. Activation of C3aR1 and C5aR1 alone with MAC insertion, mediated by aspartic protease and cysteine protease, resulting in proteolysis of synaptopodin, NEPH1, and dynamin (Figure 3) [176].

Earlier studies showed that the spontaneous remission rate of idiopathic MN is about 30%. However, who will have spontaneous remission is still a mystery [177,178,179]. Recommendations from 2021 KDIGO guidelines propose the initiation of immunosuppression therapy in more severe cases beyond the sub-classification of low risk (normal GFR, serum albumin > 3.0 g/L, and proteinuria < 3.5 g/d or proteinuria < 3.5 g/d, a decrease > 50% after six-month treatment with RAS blocker) [87]. Long before identifying PLA2R, pMN was treated with glucocorticoid and other immunosuppressants, such as alkylating agents or calcineurin inhibitors [180,181]. RCTs have shown that a combination of prednisolone and cyclophosphamide, with monthly alternation, attenuates the progression to end-stage in patients with pMN. In an early work, treating pMN with steroids or alkylating agents alone reported that the treatment did not improve renal survival in idiopathic membranous glomerulopathy, with only a slightly more complete remission with the alkylating agents [182]. Calcineurin inhibitors and CD20 antibodies have direct and indirect effects on the function of B cells. With the understanding of the pathogenesis of pMN, treatment targets focusing on B cells seem reasonable and more expected [183]. While cyclosporine does have a comparable even numerically higher remission rate than cyclophosphamide, the relapsing rate is also high [184]. Nephrotoxicity of cyclosporine is another criticized drawback. Rituximab is a rising star for treating pMN, but the most recent RCT (RI-CYCLO study) concluded that rituximab does not take advantage of a cyclic corticosteroid–cyclophosphamide regimen in neither remission rate nor adverse effects [185]. The first choice for very high risk in the sub-classification with progressive loss of kidney function remains the combination of prednisolone and cyclophosphamide by 2021 KDIGO guidelines [57]. Earlier studies reported that a high titer of PLA2R antibodies (PLA2R Ab) is associated with a poor prognosis of remission, high proteinuria production, and a high possibility of progression into end-stage renal disease [186,187,188,189]. Initiation of immunosuppressive treatment is recommended in patients with a high titer of PLA2R Ab (>50 RU/mL by ELISA) at baseline, which is a factor of high risk of progressive loss of kidney function. After six months of immunosuppressive treatment, the level of PLA2R Ab > 50 RU/mL should consider an additional course of rituximab treatment [183,190,191].

## 5. The Glucocorticoid: Still a Drug Full of Uncertainties

Glucocorticoids have been used to treat various inflammatory diseases, including asthma, allergy, several dermatologic eruptions, neuritis, and autoimmune diseases. 

Prednisolone can promote apoptosis of mature activated lymphocytes in peripheral blood to maintain immune tolerance, besides regulating the negative selection of immature T cells in the thymus [192]. In children with steroid-responsive nephrotic syndrome, treatment with 2 mg/kg/day of prednisolone leads to a significant decrease of CD4+ and CD8+ T cell counts at the first week of treatment compared to baseline and reach their lowest value at the end of the first month. The decline in B cell counts is detected later, which persists even after the cession of the prednisolone [193]. T cells are more susceptible to prednisolone; however, the reversibility is also faster with the dose tapering. The decline in B cells and B cell subtypes [CD27 (+) memory] is prolonged even after a cession of steroid treatment, which may alter antibody production in the following period.

Glucocorticoids exert their effects by binding to their receptors. The glucocorticoid receptor (GR) is a ligand-dependent transcription factor belonging to the nuclear receptor superfamily. When glucocorticoids bind to their receptors, effects may be induced by slower, classic genomic actions or more rapid, secondary non-genomic actions [194]. 

After binding and translocation into the nucleus, the glucocorticoid-GR complex influences the transcription of target genes via binding to glucocorticoid response elements (GREs) in the promotors. This binding (transactivation) can activate the transcription of phosphatase and regulatory proteins such as Mitogen-activated protein (MAP) kinase phosphatase 1 (MKP-1) [195]. The glucocorticoid GR complex may also compete against other transcription factors for binding to a site in a promotor to repress the transcription, such as activator protein-1 (AP-1), activating transcription factors (ATFs), CCAAT-enhancer binding proteins(C/EBPs), and NF-κB, to achieve transrepression [196,197]. 

The genomic effects of glucocorticoid may take a time that lags several hours. The rapid non-genomic action of glucocorticoid occurs within 60–90 min. Glucocorticoids exert non-genomic action by directly interacting with membranes, resulting in disturbance of electrolyte flux and ensuing inactivation of immune cells [198]. While interacting with the membrane of mitochondria, this may cause an increase of proton leak and decreased ATP production [199]. Non-genomic effects can also be attributed to the direct interaction of glucocorticoid-GR complex to cytosolic protein or kinases, such as the JNKs, to inhibit JNK mediated pathway [200] or depend on membrane-bound glucocorticoid receptors [201]. 

Glucocorticoids exert anti-inflammation by regulating immune responses in both genomic and non-genomic mechanisms. At cytosolic levels, via non-genomic mechanisms, glucocorticoids induce apoptosis of inflammatory cells by direct interaction with cell and mitochondrial membrane to the interference of bioenergetics and by inhibiting ERK activity [198,202]. Additionally, the glucocorticoid-receptor complex interferes with the activity of AP-1, a pro-inflammatory transcription factor consisting of c-Fos and c-Jun, by direct protein–protein interaction. Meanwhile, glucocorticoids also depend on transactivation and transrepression to conduct genomic anti-inflammatory activities. For example, to repress the transcription activity of AP-1, the glucocorticoid receptor complex reduces the mRNA level of c-Jun at AP-1 sites of c-Jun promotor by transrepression. Alternatively, glucocorticoid induces MKP-1 to inactivate JNK and the following c-Jun transcriptional activity by transactivation [200,203,204]. Likewise, the glucocorticoid-GR complex also represses NF-κB by direct transrepression or induces IκB synthesis, via transactivation, to tether NF-κB to form an inactive complex in the cytoplasm [205]. Direct interaction between the GR and RelA protein of NF-κB, or by mutually competing for a limiting co-factor, CREB binding protein at cytosolic levels also contributes to glucocorticoid’s anti-inflammatory effect [206,207,208]. Furthermore, by the increased expression of tristetraprolin (TTP), an mRNA destabilizing protein, glucocorticoid-GR complex promotes the degradation of the pro-inflammatory cytokine mRNA, i.e., the mRNA of IL-8 [209]. 

Although using glucocorticoid mainly suppresses inflammation, glucocorticoid is also a stress hormone. As inflammation is a necessary protective mechanism, treatment with glucocorticoids sometimes leads to pro-inflammatory effects. For example, glucocorticoids, such as dexamethasone and cortisol, are known to enhance the expression of TLR2 in human keratinocytes after *Propionibacterium acnes* infection or stimulated by TNF-α, or IL-1α [210]. Dexamethasone also induces NLRP3 messenger RNA and protein to enhance ATP-mediated cytokine release, including mature IL-1β, TNF-α, and IL-6 [211]. Prednisolone is also known to induce alternative macrophage activation and affect macrophage polarization to M2 predominant phenotype and fails to ameliorate mesangial proliferative glomerulonephritis in an animal model [212]. 

Nonetheless, sensitivity to glucocorticoids varies among individuals. Polymorphisms of the glucocorticoid receptor gene NR3C1 may cause different glucocorticoid sensitivity and resistance. For example, ER22/23EK polymorphism is associated with glucocorticoid resistance [213]. Alternative RNA splicing, alternative translation, and post-translation modification are known to generate different GR isoforms. The glucocorticoid receptor isoform α is the most abundant isoform and the primary mediator of glucocorticoid action, while the isoform β may inhibit glucocorticoid activity [214]. Lower ratio of GRα/GRβ often correlates with the resistance [215]. The expression of isoform β may result in the formation of α/β heterodimers that decrease glucocorticoid sensitivity [216]. Furthermore, increased expression of GRβ is promoted by pro-inflammatory cytokines or immune activators [217]. Post-translation modification of GR also regulates the functions of receptors. For example, deacetylation of the glucocorticoid-GR complex by histone deacetylase-2 (HDAC2) is necessary for repressing transcriptional signaling of NF-κB [218]. The diversity of isoforms as a whole delivers a complement and composite tissue-specific phenotype to every individual [219]. 

Several molecular mechanisms of glucocorticoid resistance have now been elucidated. The p38 MAP kinases suppress the glucocorticoid GR complex function by directly tethering the GR complex ligand-binding domain. JNK kinases and Mitogen-activated protein kinases/extracellular signal-regulated kinase (MAPK/ERK) inhibit transcription activity of glucocorticoid GR complex by phosphorylation. These may result in glucocorticoid resistance in chronic inflammatory conditions [220,221]. Mutual antagonism with crosstalk exists between glucocorticoid GR complex and TNF-α-NF-κB/AP-1 axis. Perturbation of balance may contribute to the resistance of glucocorticoid [217,222,223,224]. As mentioned above, reduced histone deacetylase-2 (HDAC2) expression may also lead to glucocorticoid insensitivity for anti-inflammation [218]. Raised macrophage migration inhibitory factor, a pro-inflammatory factor induced by glucocorticoid, also leads to glucocorticoid resistance in experimental autoimmune encephalomyelitis and systemic lupus erythematosus [225,226]. Increase of the product of multidrug resistance– 1 gene (MDR1), P-glycoprotein, is known to mediate drug efflux [227,228]. Therefore, the polymorphism of the P-glycoprotein gene also contributes to glucocorticoid resistance [229].

Nonetheless, inadequate dose or duration owing to the narrow therapeutic window and associated toxicity of glucocorticoids may cause ineffectiveness of the treatment. Therefore, some approaches are underway to maximize the benefit to risk ratio. For example, one method is to tweak the conventional glucocorticoid optimally with a long-circulating polyethylene glycol liposomal carrier system, which promotes the accumulation in tissue and decreases plasma concentration simultaneously [230,231,232]. Most adverse effects with the glucocorticoid are claimed from the transactivation [233]. Based on the concept, new selective glucocorticoid receptor agonists (SEGRAs) characterized with dissociation of transactivation from transrepression are under development [234,235]. The least is to establish a steroids-sparing regimen which has been a trend in medical fields, to avoid the side effects. 

Besides anti-inflammatory effects, glucocorticoids directly affect podocytes of nephrotic syndrome, including phosphorylation of nephrin, maintenance of nephrin expression, stabilization of cytoskeleton, and prevention of apoptosis. Tyrosine phosphorylation of nephrin is essentially required to stabilize and restore foot process architecture and podocyte survival status [72]. Dexamethasone is known to resist angiotensin II-induced podocyte injury via increasing nephrin phosphorylation by Fyn/Nck complex, an Src family tyrosine kinase, in vitro [236]. As the mutant signaling transient receptor potential channel 6 (TRPC6) results in an overexpressed nuclear factor of activated T cell (NFATc-1), which cause steroid-resistant nephrotic syndrome and glomerulosclerosis in vivo, the zinc-finger transcription factor Krüppel-like factor 15 (KLF15) binding to the promotor of NFATc-1 increases expression of both nephrin and podocin to ameliorated podocyte injury [237,238]. Dexamethasone can increase the expression of KLF15, stabilize the expression of TRPC6, and block associated signal pathways on podocytes [239,240]. In a rat model of puromycin aminonucleoside nephrosis, dexamethasone can increase polymerized actin and activity of the actin-regulating GTPase Rho A to stabilize actin filaments [241]. An in vitro study showed that glucocorticoids decreased the activity of Ras-released C3 botulinum toxin substrate 1 (Rac1, a Rho GTPase). Overexpression of Rac1 leads to nephrotic syndrome with minimal change disease-like appearance in kidney biopsies in animal models [242,243]. Dexamethasone also decreases p53 expression in PAN mice via stabilizing the PI3K/Akt signal pathway to inhibit podocyte apoptosis [244,245].

Steroids are still the mainstay drug for the treatment of glomerular disease. However, barriers such as predicting resistance, avoiding accumulating toxic effects, and decreasing relapse are still waiting to be conquered. Integrating available or new biomarkers with clinical random control studies to develop new medication and therapeutic strategies may someday achieve these goals.

## 6. Cyclophosphamide, Cyclosporine, and Mycophenolate Acid: Indispensable Helpers

### 6.1. Cyclophosphamide

Cyclophosphamide (CYC), an alkylating drug, was first used for cancer treatment and later in treating connective tissue diseases and immune-mediated nephritis. It is an inactive prodrug that is converted by the liver enzyme p450 to 4-hydroxycyclophosphamide, and undergoes metabolism to several intermediates with alkylating activity to interfere with DNA replication and transcription of RNA. The primary metabolites are phosphoramide mustard and inactive acrolein [246]. Phosphoramide mustard is further metabolized to produce nornitrogen mustard, which also has alkylating activity [247]. The intermediate metabolite, 4-hydroxycyclophosphamide, is converted to a non-cytotoxic compound carboxy-phosphamide by aldehyde dehydrogenases. There is a large individual variability in the pharmacokinetics and metabolism, dependent on polymorphism of p450 and the existence of aldehyde dehydrogenase [248,249,250].

The active metabolites with alkylating ability crosslink guanine residue in DNA, which leads to cell apoptosis. Unlike glucocorticoids which primarily suppress T cells, both T cells and B cells are sensitive to cyclophosphamide, and B cells are reduced first [251]. After administration, the nadir of leukocyte count is observed around the 8th–14th day, and the counts recovered about 25 days later. High expression of aldehyde dehydrogenase in the Treg may contribute to resisting cyclophosphamide [252]. Besides this, high-dose cyclophosphamide in an immunosuppressed rat model can lower CD103+ dendritic cell numbers and modify the expression of surface markers on this cell subset. That may further reduce antigen uptake capacity but enhance the capacity to prime CD4+ cells. Via the TLR/MyD88/MAPK pathway, high dose cyclophosphamide increased Treg and reduced the Th1/Th2 polarization and Th17 subset [253]. This modulation in T cell subsets may play a role in treating autoimmune and chronic inflammatory diseases.

Like glucocorticoid, cyclophosphamide has a narrow therapeutic index. Besides the infectious risk, bladder toxicity with gross hematuria, gonadal toxicity, and increased risk of lymphoma, leukemia, and bladder cancer make cyclophosphamide notorious. Nonetheless, cyclophosphamide is indispensable. Cyclophosphamide is almost ubiquitous in the treatment of immune and inflammation-mediated nephropathy. Furthermore, it is widely applied in steroid-resistant-minimal change disease, combining steroid, ANCA-associated vasculitis, and lupus nephritis as induction therapy [87].

### 6.2. Calcineurin Inhibitors

Calcineurin is an essential calcium-dependent phosphatase for T cell functions. There are two main isoforms: the α-isoform is essential for kidney development, while the β-isoform has a predominant role in the immune system. This difference may contribute to the nephrotoxicity of calcineurin inhibitors [254].

The immunosuppressive effect of the calcineurin inhibitors is to block calcineurin-mediated dephosphorylation of nuclear factor of activated T cells (NFAT) signaling in T cells and lead to a decrease in the production of IL-2 and other lymphokines from T cells. The recruitment of cytotoxic T cells is therefore attenuated [255]. Cyclosporine A(CsA), associated with intracellular binding protein(cyclophilins) to inhibit calcineurin activity, is the prototype of calcineurin inhibitors. CsA also inhibits the activation of JNK and p38 signaling pathways, which are triggered by antigen recognition via T cell receptor and CD28 costimulatory receptor. CsA is thus a highly specific inhibitor of T cell activation [255]. Nonetheless, hypogammoglobulinemia and B cell hypo-responsiveness can be observed in the treatment with CNI. Besides T cell dependent mechanism, CsA directly interferes with B cell migration by disrupting the O_2_ sensing molecular switch, destabilizing HIF-1α, and preserving responsiveness of B cells to C-X-C Motif Chemokine Receptor 4 (CXCR4). Restoration of the hypo-responsiveness to CXCR4 disrupts the coordinated localization of B cell in dark zones and light zones of germinal center and suppresses B cell response [256]. Tacrolimus is another, more potent CNI with different intracellular binding proteins (i.e., FK binding protein), associated with less nephrotoxicity [257]. 

CNI treats immune-mediated nephropathy, mainly mediated by its immunosuppressive action. Nonetheless, calcineurin inhibitors can treat podocytes as a direct target. Rho GTPase plays a role in cytoskeletal rearrangement of cellular process, cell motility/migration, polarization, and cell cycle progression. Whether up-regulation of Rho A or inhibition has similar adverse effects on glomerular filtration barrier function and un-stabilized expression of Rho A results in proteinuria. Synaptopodin inside the podocytes is a regulator of Rho GTPase, stabilizing the expression of Rho A [258,259]. Dephosphorylation of synaptopodin mediated by calcineurin leads to loss protection from the interaction with 14-3-3β protein, and synaptopodin becomes susceptible to cathepsin L mediated degradation. CsA can protect synaptopodin from dephosphorylation and ensuing cathepsin L–mediated degradation, which leads to a direct antiproteinuric effect with stabilization of actin cytoskeleton [260].

### 6.3. Mycophenolate Mofetil/Mycophenolic Acid Analogue

Mycophenolate mofetil is a prodrug that is hydrolyzed in the blood to mycophenolate acid (MPA), an inhibitor of inosine 5′-monophosphate dehydrogenase (IMPDH). This dehydrogenase is the rate-limiting enzyme with two isoforms, IMPDH1 and IMPDH2, for de novo GTP synthesis [261]. Adequate GTP synthesis is vital for lymphocyte proliferation, depending on adenosine deaminase (ADA) and inosine 5′-monophosphate dehydrogenase (IMPDH). As other cells can also adopt with salvage pathway, lymphocytes sorely depend on de novo synthesis to maintain an adequate level of GTP. Unlike IMPDH1, which is expressed constitutively in lymphocytes, IMPDH2 is inducible and highly expressed in T and B cells after mitogenic stimulation or viral transformation [262,263]. MPA inhibits the IMPDH2 more potently (4.8 times) than IMPDH1. This selectivity of MPA mitigates the toxicity resulting from inhibition of the constitutive IMPDH1 in cells other than lymphocytes [264]. MPA is suitable as an adjunct to other immunosuppressants by conserving the activation-induced cell death (AICD) [264,265]. Similarly induced apoptosis of monocytes/macrophages and monocyte-derived dendritic cell with correlated downregulation of co-stimulating factors and adhesion molecules (CD40, CD54, CD80, and CD86) are also found [266,267]. Depletion of guanosine nucleotides and GTP by MPA reduces the expression of adhesion molecules such as VLA-4 and LFA-1, leading to a decrease in adhesion and diapedesis of CD4+ and CD8+ T cells [268]. By depletion of guanosine nucleotides and GTP, MPA can reduce intracellular levels of tetrahydrobiopterin (BH4), an essential co-factors of inducible NOS (NOS2). MPA, therefore, can suppress the NOS2 mediated NO production in the inflammatory pathway [269,270]. 

Like suppressing lymphocytes and monocytes, MPA also inhibits the proliferation of mesangial cells and mesangial matrix expansion [271]. Furthermore, proteinuria induces ER stress by mitochondrial dysfunction associated with ATP depletion. This ER stress causes mislocalization of nephrin to damage podocytes. Conversely, inhibition of the de novo synthesis pathway restores intracellular ATP level via salvage pathway in podocytes, which corrects the post-translation processing of nephrin and maintains podocyte homeostasis [272]. MMF is also known to reduce urokinase receptor (uPAR) signaling, which leads to podocytopathy and foot process effacement in podocytes of a lupus nephritis animal model [273,274].

## 7. Rituximab and Complement Inhibitors: The Rising Stars

### 7.1. Rituximab

Rituximab is a mouse–human chimeric antibody, targeting B cells with CD20. It has been used in treating leukemia, lymphoma, and rheumatoid arthritis with inadequate response to TNF antagonist therapy [275]. The first success in treating immune-mediated kidney disease by Rituximab was demonstrated by a pilot trial to treat idiopathic membranous glomerulonephritis in 2008 [276]. The mechanism by which rituximab eliminates B cells is binding to CD20 on the surface of B cells, with the exposure of the Fc portion, leading to the antibody or complement-dependent cytotoxicity to B cells [277]. Rituximab depletes B cells and affects T cell function since B cells can present antigens to activate T cells [278]. Rituximab is known to suppress Th17 immunity via SMPDL/ASM [51,52]. Escalation of a subpopulation of Treg (FoxP3+) or their functions following the depletion of B cells may also benefit the therapeutic values of rituximab [279]. 

Another hypothesis for treating nephropathy by Rituximab is a non-immune mechanism, which involves directly targeting podocytes. Rituximab can affect sphingomyelin phosphodiesterase acid-like 3b(SMPDL-3b) protein on the podocytes. Podocytes in patients with FSGS have high motility owing to the suPAR associated αVβ3 integrin activity. SMPDL3b decreases the binding between suPAR and β3 integrins to prevent actin-cytoskeleton remodeling. Rituximab prevents the downregulation of SMPDL-3b and ASMase in podocytes, induced by sera of the patient with recurrent FSGS [280]. 

The 2021 KDIGO guidelines recommend rituximab to treat minimal change disease, idiopathic membranous glomerulonephritis, ANCA-associated vasculitis, and second-line therapy of lupus nephritis. However, as an autoantibody and immune complex associated disease, treatment to IgAN by Rituximab seems ineffective. This refractory response is attributed to gd-IgA secreted by plasma cells with low amounts of CD20 [140,141].

### 7.2. Complement Inhibitors

Another inspiring category of medication for the glomerular disease is complement inhibitors. Although some glomerular diseases are related to the immune response mediated dysregulating complement system activation, these complement inhibitors may help treat the disease. For example, C5a receptor 1, a complement receptor, regulates the dendritic cells and is essential to T cell immunity. Interacting with C5a leads to T-cell mediated anti-MPO glomerulonephritis with associated neutrophil recruitment and Redox reaction [281]. The medications to inhibit C5a associated injury have been shown to ANCA-associated vasculitis and atypical hemolytic uremic syndrome (aHUS). For example, Eculizumab, a recombinant monoclonal antibody, prevents cleavage of C5 into C5a and C5b for the treatment of aHUS with progress in a phase IV study [282]. Another oral C5a inhibitor, Avacopan, has just finished its phase III trial, with a better sustained remission rate than corticosteroid at week 52, and obtained the U.S.FDA approval in the maintenance treatment of ANCA associated vasculitis [283]. There are several ongoing trials of complement inhibitors in the treatment of glomerular diseases, including agents for novel C5a inhibitor (NCT03841448), C3 inhibitor (NCT03453619), MASP 2 inhibitor (NCT02682407), factor B inhibitor (NCT03373461), and factor D inhibitor (NCT03832114).

## 8. Nrf2 Activator: Accessory Agents for Immunosuppressants

Nuclear factor erythroid 2-related factor-2(Nrf2) activator, an anti-oxidant, has been shown to ameliorate steroid-resistant lupus nephritis in an animal model [284]. In an inflammatory state, pro-inflammatory transcription factors, such as NF-κB and AP-1, activate the transcription of inflammatory cytokines and chemokines genes. Glucocorticoids can reverse this pro-inflammatory state by rewinding and compacting the chromatin via the recruitment of HDAC2 [285]. However, decreased expression of HDAC2 may depreciate the anti-inflammatory action of glucocorticoids [218]. It is known that a decreased expression of histone deacetylase 2 (HDAC2) is associated with the resistance to glucocorticoids. This condition has been found in pediatric steroid-resistant nephrotic syndrome [286]. Although Nrf2 activator cannot replace glucocorticoids totally for treating immune-mediated and inflammatory disease, it has been used to enhance the efficacy of glucocorticoids. As Nrf2 activators increase the expression of HDAC2, thus reducing the resistance to glucocorticoids [287,288]. It works much similar to the idea of channel ushering drugs in traditional Chinese herbal medicine to change the micro-milieu and guide the dominant drug to the channel. Drugs with different foci in the pathogenesis may be complementary to each other.

## 9. Conclusions

**Figure 4:**
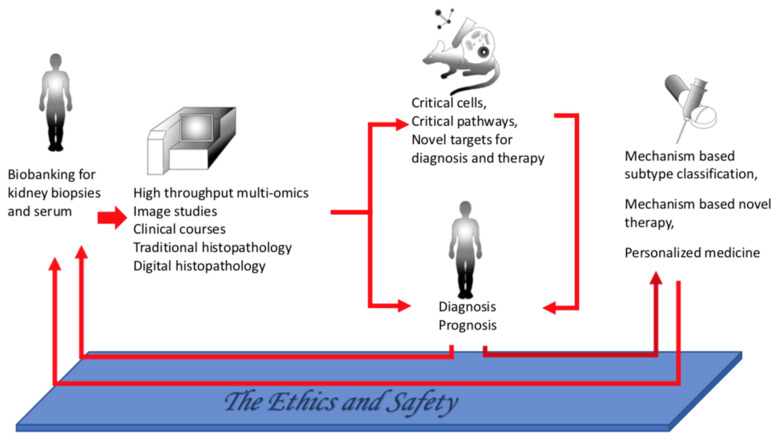
A paradigm of precision medicine in the field of glomerular diseases.

Although with the advancement of molecular biology and various high throughput technologies, more and more of the pathogenesis underlying each glomerular disease and the operating mechanism of various drugs and immunosuppressants are understood, there are still many obstacles. For example, based on existing disease classifications, we even now cannot make flawless decisions about which drug is the best choice. Furthermore, there is still a trial-and-error model of treatment that leaves patients suffering from unnecessary side effects due to ineffective medical treatments, and the relapse of the disease is very troublesome. Fortunately, with the rapid advancement of technology and the spread of the concept of precision medicine (Figure 4), just like the discovery of PLA2R antibodies, we can proactively look for more suitable and novel biomarkers, critical cells or pathways in the diseases, and accordingly develop more suitable and adequate treatment modalities. This precision medicine can shorten the time to explore and will help accelerate this trend. Therefore, it is hoped that we can have disease classification and diagnosis customized according to the mechanism of disease and can provide appropriate treatment in the near future. Ultimately, the ideal personalized medicine can then be achieved.

## Data Availability

Not applicable.
